# Tuning wettability and electrical conductivity of single-walled carbon nanotubes by the modified Hummers method

**DOI:** 10.1038/s41598-022-08343-5

**Published:** 2022-03-14

**Authors:** Grzegorz Stando, Sujie Han, Bogumiła Kumanek, Dariusz Łukowiec, Dawid Janas

**Affiliations:** 1 Department of Organic Chemistry, Bioorganic Chemistry and Biotechnology, Faculty of Chemistry, Silesian University of Technology, B. Krzywoustego 4, 44-100 Gliwice, Poland; 2 Department of Chemistry, University of Pittsburgh, Pittsburgh, PA USA; 3 School of Materials Science and Chemical Engineering, Ningbo University, 818 Fenghua Road, Ningbo, People’s Republic of China; 4 Laboratory of Material Engineering and Environment, KOMAG Institute of Mining Technology, 44-101 Gliwice, Poland; 5 Institute of Engineering Materials and Biomaterials, Faculty of Mechanical Engineering, Silesian University of Technology, Konarskiego 18, 44-100 Gliwice, Poland

**Keywords:** Carbon nanotubes and fullerenes, Electronic properties and materials

## Abstract

Partial oxidation of nanocarbon materials is one of the most straightforward methods to improve their compatibility with other materials, which widens its application potential. This work studied how the microstructure and properties of high crystallinity single-walled carbon nanotubes (SWCNTs) can be tailored by applying the modified Hummers method. The influence of temperature (0, 18, 40 °C), reaction time (0 min to 7 h), and the amount of KMnO_4_ oxidant was monitored. The results showed that depending on the oxidation conditions, the electronic characteristics of the material could be adjusted. After optimizing the parameters, the SWCNTs were much more conductive (1369 ± 84 S/cm with respect to 283 ± 32 S/cm for the untreated material). At the same time, the films made from them exhibited hydrophilic character of the surface (water contact angle changed from 71° to 27°).

## Introduction

Carbon nanotubes (CNTs) and graphene have a unique combination of properties, which has kept them at the forefront of research for the past decades. Their electrical^1^, thermal^2^, and optical^3,4^ characteristics are not only tunable, but upon optimization, they may surpass those of many traditional materials. For instance, the current density of individual single-walled carbon nanotubes (SWCNTs) approaches 4 × 10^9^ A cm^−2^, outperforming copper by three orders of magnitude^5^. What is more, the thermal conductivity of graphene monolayers could exceed over 5000 W m^−1^ K^−1^, an order of magnitude improvement over copper again^6^. As a consequence, they are envisioned to become key components of many technologies in the upcoming future.

Unfortunately, macroscopic ensembles made from these materials in the form of films or fibers^7^ exhibit lower performance. Regarding electrical conductivity, this problem is partially caused by the issues with charge propagation at the interface of individual building blocks (single CNTs or graphene flakes). These constituents are often misaligned and separated by air-filled cavities, which are electrically insulating. To alleviate this problem, carbon nanostructures are often combined with other conductive materials like conductive polymers^8–10^ or metals^11,12^, which can facilitate the energy transfer from one ensemble element to the other. However, this solution requires proper integration of the host (nanocarbon) with the guest (conductive additive), which is challenging. Carbon nanomaterials have relatively inert surfaces under typical conditions, provided that the material is free of imperfections. Therefore, special measures must be taken to make them compatible with other materials. The popular solution to this problem is chemical functionalization, which can introduce hydrophilic groups on the surface, thereby increasing the affinity of a broad spectrum of materials to nanocarbon^13,14^.

Most commonly, grafting of CNTs begins with partial oxidization of the material. A myriad of oxidizing agents can be employed, such as a mixture of HNO_3_ and H_2_SO_4_^15–17^, KMnO_4_^18^, H_2_O_2_^19,20^_,_ O_3_^21^, piranha solution^22^, concentrated^23^ or fuming nitric acid^24^, etc. The microstructure and chemical composition of the product is then dependent on the purity of the substrate and the severity of the engaged oxidation conditions^25^. The treatment proceeds through four distinct stages: doping, which is observed only for selected oxidizing mixtures (insufficient amount of oxidant to implant the functional groups), functionalization (optimum conditions to graft), oxidative unzipping (excessive oxidation causing the deterioration of the native structure of the material), and, finally, combustion to CO_2_. Drastic changes to the topology of the nanocarbon networks resulting from these transformations are reflected by significant alteration of the electrical conductivity of the CNTs. Previously, we observed that when MWCNTs are exposed to fuming HNO_3_, the system's resistance decreases by half, while the vapors of H_2_O_2_/H_2_SO_4_ (piranha solution) or O_3_ notably increase the resistance^21^. In the highlighted cases, doping and chemical modification were predominant, respectively. Brønstead acids such as HNO_3_ and H_2_SO_4_, when employed at low temperature, can considerably dope the CNTs^26^. In such a case, in the absence of defects, the extent of oxidation will be negligible. Consequently, even a simple immersion of a CNT ensemble in HNO_3_ solution improves its electrical conductivity. Pareth et al. observed a 33% decrease in resistance caused by dipping CNTs in 70% HNO_3_ for 3 h at room temperature^27^. Furthermore, Geng and colleagues also showed that the CNT resistance decreases by 40% after 12 M HNO_3_ treatment at room temperature for 60 min^28^. However, when more vigorous conditions are employed, even CNTs of high crystallinity lose immunity to oxidation.

Regarding the oxidation mechanism, one needs to consider that during the oxidation of CNTs, the oxygen-containing functional groups grafted on the surface may also be oxidized themselves. Consequently, after the process, the CNT side-wall is often equipped with various functionalities such as carboxyl, formyl, hydroxyl, ether, carbonyl, acid anhydride, etc^29^. The distribution of these groups determines how hydrophilic a network made from such oxidized CNTs will be. Gerber and co-workers showed the impact of oxidation time on the ratio between particular oxygen groups. For example, in the early stage of the reaction using HNO_3_ as the oxidant, at the temperature of 120 °C, the carbonyl and hydroxyl groups prevailed. Later, as the process was continued, the content of these groups started to decrease due to the oxidation of these species. At this point, the authors observed the formation of lactones, acid anhydrides, and carboxylic acid groups on the CNT surface^29^. A similar effect can be obtained by increasing the oxidation temperature, which speeds up the kinetics of the underlying chemical transformations^15,22,23,29–31^. Besides dynamic changes to the chemical composition, the product's microstructure is often severely affected. In the case of MWCNTs, oxidation gradually reduces the number of walls^32,33^. Therefore, if SWCNTs (or MWCNTs under sufficiently harsh environments) are employed, a similar treatment can cause unzipping of the tubes, eventually giving graphene oxide nanoribbons (GONRs)^34–37^.

The popular Hummers method used to produce graphene oxide (and subsequently reduced graphene oxide upon reduction) is a convenient tool for studying the oxidation of CNTs. Dimiev et al. demonstrated how the ratio between MWCNTs and KMnO_4_ controls whether oxidation or unzipping dominates. The reaction was not successful for the MWCNTs/KMnO_4_ ratios of 1/0.06 and 1/0.12, while increasing the oxidant amount to 1/0.5 and 1/1 enabled the oxidation of MWCNTs^36^. To our surprise, a literature search revealed that the oxidation process of CNTs by this technique was not investigated in detail using SWCNTs, which could shed more light on the mechanism of their oxidative degradation.

In this article, high-quality SWCNTs were doped/oxidized under the conditions of the modified Hummers employing KMnO_4_ and H_2_SO_4_. We varied the time, temperature, and the relative amount of the KMnO_4_ oxidant to SWCNTs to (i) study the oxidation phenomenon and (ii) tailor the properties of the modified SWCNTs. The reaction progress was monitored by Raman spectroscopy, while the product's microstructure was visualized by Scanning Electron Microscopy (SEM). High-doping and oxidation capabilities of the reaction system enabled us to tune the electrical properties and hydrophilicity of the material by appropriate selection of the operational conditions.

## Experimental

### Oxidization of SWCNTs

SWCNTs (Tuball™, OCSiAl, Luxembourg) were oxidized by the modified Hummers method^38,39^. Three parameters of the reaction were varied: time, temperature, and the amount of KMnO_4_ oxidant (POCH, Poland). Samples were collected for Raman spectroscopy after executing the reaction for the specified duration: 0 min (reaction quenched immediately upon introducing KMnO_4_), 10, 20, 30, 40, 50, 60, 120, 180, 240, 300, and 420 min. Three reaction temperatures were chosen: 0 °C, 18 °C, and 40 °C. The following ratios of KMnO_4_ to SWCNTs were used: 0 (exposure of SWCNTs to the solution of H_2_SO_4_ and H_3_PO_4_ in the absence of KMnO_4_), 0.15, 0.25, 0.45, 0.95, 1.9, 3.75, and 7.5.

0.2 g of SWCNTs were added into the mixture of 120 mL 95% H_2_SO_4_ (Chempur, Poland) and 12 mL 85% H_3_PO_4_ (Chempur, Poland). The mixture was stirred mechanically for 30 min at 300 rpm (OST Basic yellow, IKA Works, USA) while KMnO_4_ was dosed continuously. Then, the reaction progressed for the time specified above, counted from the completion of KMnO_4_ addition. Next, the reaction mixture was quenched in 400 mL of distilled H_2_O and 20 mL of 30% H_2_O_2_. Then, the solid material was separated by vacuum filtration using PTFE membranes (diameter 47 mm, pore size: 0.22 µm; Fisherbrand, Canada). The crude product was purified using 100 mL of 10% HCl, 100 mL of distilled water, and finally 100 mL of methanol. The obtained material was dried at 80 °C overnight to remove moisture introduced during the work-up.

### Manufacture of films from oxidized SWCNTs

The nanocarbon films were produced by a wet method described by us previously^40^. In short, SWCNTs, ethyl cellulose (EC; binder), and acetone/toluene mixture (1:1 by weight) were combined and sonicated (Hielscher UP200St, Germany) at 100% amplitude over an ice bath until homogenous dispersion was observed. Next, the dispersion was deposited onto a Nomex^®^ sheet by drop-casting. After drying, a free-standing film was produced, from which the EC binder was removed by thermal annealing in air^22^.

### Characterization of oxidized SWCNT materials

The reaction progress was analyzed using:

- Evolution of the intensity of characteristic peaks for nanocarbon materials indicative of the chemical state of the SWCNT surface was gauged by Raman spectroscopy (Renishaw, Germany). The samples were evaluated at 10 random areas (laser wavelength 514 nm, laser power 5%, wavenumber range 1000–3000 cm^−1^, exposure time 10 s, and accumulation number 3) to verify the homogeneity of oxidation. I_D_/I_G_ ratios were determined by dividing the areas of the corresponding peaks as this approach gives more reliable results^41^. In addition, the samples obtained from treatments at 0 °C were characterized from 100 to 3000 cm^−1^, which enabled analysis of the RBM area.
- X-ray photoelectron spectroscopy (XPS) measurements were conducted using 128 element multichannel plate (MCP) detector coupled with ESCALAB 250Xi XPS apparatus (Thermo Fisher, USA). The detection conditions were as follows: source gun type—Al Kα (1486.6 eV), plot size—650 µm, pressure of the analysis chamber—8 × 10^–9^ Pa. Spectra in C1s and O1s areas were recorded at energy pass—50.0 eV, energy step size—0.100 eV, number of scans—5. Thermo Scientific Avantage software was used for deconvolution and analysis.
- Morphology of the oxidation products was characterized by High-Resolution Scanning Electron Microscopy (HR SEM, SUPRA 35 ZEISS, Germany). The materials were investigated at 8 kV.
- Electrical conductivity of thin free-standing nanocarbon films made of the reaction products was measured by the 4-probe method (Keithley 2182A source meter, USA). The current used during the measurement was 100 mA, which was low enough not to warm the sample by Joule heating.
- Water contact angles were determined by a custom-made setup equipped with a CMOS camera (Thorlabs, USA). First, 10 μL of demineralized water was deposited onto the selected samples, after which an image was recorded with the camera equipped with a macro lens. The temperature and humidity during the measurements were 21 °C and 53%, respectively. ImageJ software (Contact angle plugin) was used for analysis.
- Thermoelectric properties of the samples were registered using a custom-made thermopower setup (LBR CAMSEEB, Poland), which measured Seebeck coefficients in a temperature range from 40 to 90 °C. More details about measurements can be found in our earlier contribution^42^.

## Results and discussion

### Characterization of oxidized SWCNTs

**Figure 1:**
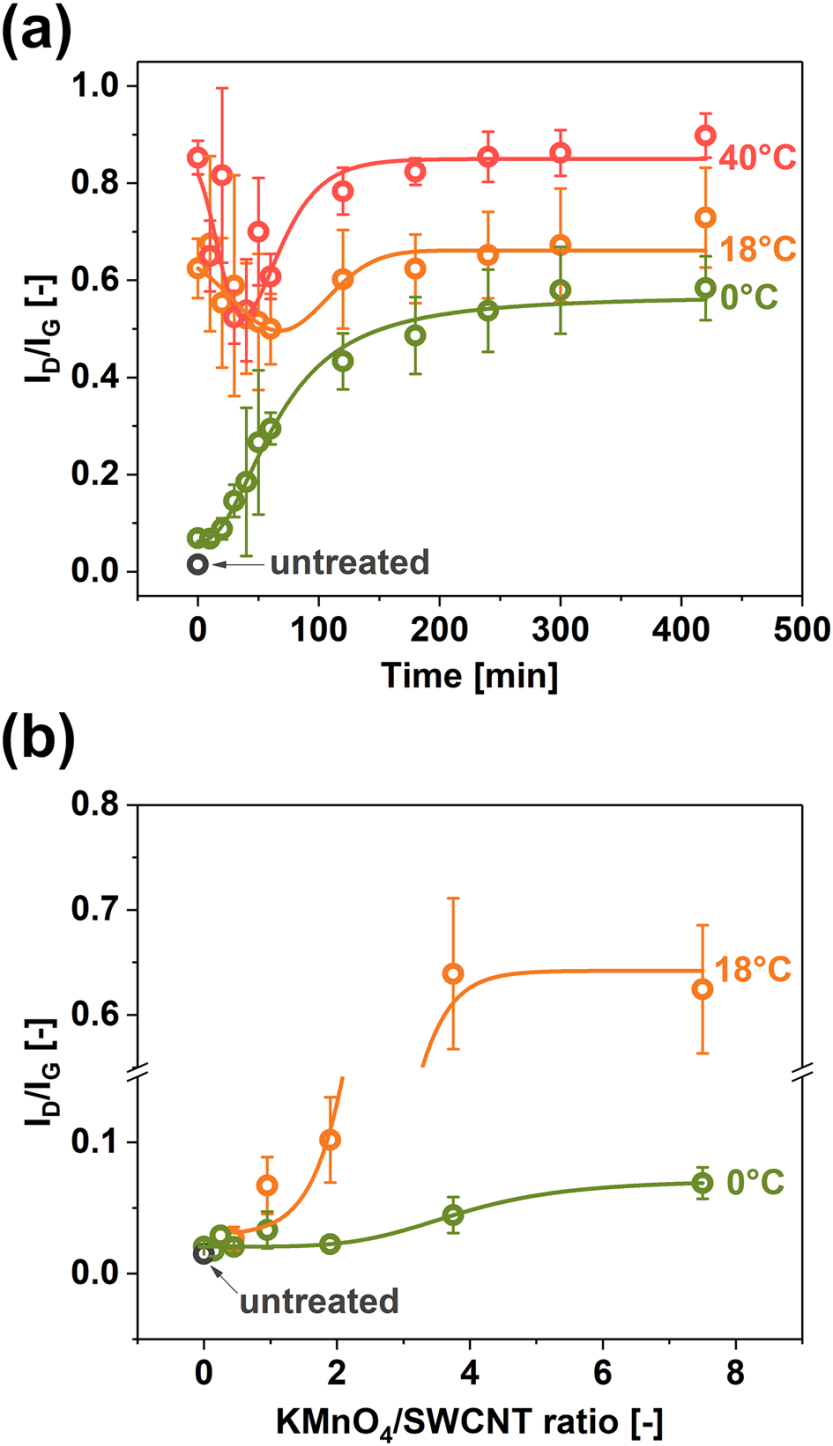
The impact of SWCNT oxidation on the I_D_/I_G_ ratios of the obtained products determined by Raman spectroscopy as a function of **(a)** time (KMnO_4_/SWCNTs = 7.5), **(b)** KMnO_4_/SWCNT ratio (t = 0 min).

The I_D_/I_G_ ratio was used to quantify the influence of the oxidation conditions on the SWCNTs. The D band reflects the level of imperfection in the SWCNTs in the form of sp^3^-hybridized carbon atoms, which can be used to gauge the extent of chemical modification^43^. Therefore, one may expect an increase in this ratio as the oxidation progresses due to the growing content of defects. The evolution of the D peak intensity was studied for three reaction temperatures of 0 °C, 18 °C, and 40 °C (Fig. 1a). In these experiments, the KMnO_4_/SWCNT ratio was 7.5.

Pure SWCNT material had a low I_D_/I_G_ value of 0.015 ± 0.001, which indicated its high crystallinity (Fig. 1a). Once the SWCNTs were exposed to oxidizing conditions, a considerable increase in the I_D_/I_G_ ratios was observed. While the treatment conducted at 0 °C increased the I_D_/I_G_ ratio to only 0.069 ± 0.012, it was as high as 0.624 ± 0.061 and 0.853 ± 0.034 when the temperature was elevated to 18 °C and 40 °C, respectively.

Investigation of the kinetics showed two treatment pathways, which differed for the reactions carried out while cooling the mixture (0 °C) and at the temperatures of 18 °C and 40 °C. In the former case, the reaction proceeded steadily up to 60 min and then gradually decelerated. After 420 min, the I_D_/I_G_ ratio reached the level of 0.58 ± 0.07, showing the introduction of a significant level of disorder to the SWCNTs.

Interestingly, for the treatments at 18 °C and 40 °C, the process took a different course. After the initial steep rise of the I_D_/I_G_ ratios compared with the pure SWCNTs, a decrease in this parameter was observed, which is visualized in the plot as local I_D_/I_G_ ratio minima. We hypothesize that the harsh processing conditions preferentially oxidize the small-diameter population of the SWCNT mixture^44^, which contains a broad spectrum of diameters ranging from 0.75 to 3.0 nm^45^. In this scenario, the combustion of these species to CO_2_ is more rapid than the introduction of the oxygen functional groups into the rest of the SWCNT population. This may explain why the amount of disorder in the system initially decreased. Then, upon removing these small-diameter SWCNTs, only the increase in the I_D_/I_G_ ratio was observed indicative of progressive functionalization of the species of larger diameter.

To validate this suspicion, we used Raman spectroscopy to analyze how the shape of the Radial-Breathing Mode (RBM) is affected by the treatment (Fig. S1). At the selected excitation wavelength of 514 nm, two populations of SWCNTs can be discerned for the untreated material with peak maxima at 146 and 176 cm^−1^. These values correspond to SWCNTs with mean diameters of 1.61 nm and 1.34 nm. Then, after only 10 min of the process, the latter decreased in intensity considerably. In this case, the maximum intensity was recorded for 151 cm^−1^, which translates to SWCNTs of 1.55 nm in diameter. Hence, the mean diameter of the SWCNTs in the sample was increased (SWCNTs in the small-diameter were apparently eradicated). Lastly, 180 min of oxidation under the specified conditions silenced out the RBM mode altogether, suggesting that substantial changes in microstructure occurred.

Furthermore, when 420 min of oxidation were reached, the I_D_/I_G_ amounted to 0.73 ± 0.10 and 0.90 ± 0.05 for 18 °C and 40 °C treatments, respectively (the relatively large error bars to the values of I_D_/I_G_ quotients result from the polydispersity of SWCNTs employed for the study). The oxidation of the material at the higher temperature introduced more oxygen-containing functional groups or promoted their oxidation to moieties such as carboxyl group.

To better understand the oxidation mechanism by the modified Hummers method, we decided to study how different ratios of KMnO_4_ to SWCNTs affect the crystallinity of the product (Fig. 1b). The reaction was quenched immediately after finishing the introduction of the oxidant hence the notation of t = 0 min. As specified in the Experimental section, the actual reaction time was 30 min at this point since KMnO_4_ was added gradually for half an hour in every case*.* The temperatures of 0°C and 18°C were selected for this analysis since the kinetics of the process would be too fast at 40 °C. As expected, the higher the oxidant amount, the more disorder was introduced into the material. The influence of KMnO_4_ on the chemical modification was mild at 0 °C. The I_D_/I_G_ ratios increased from 0.015 ± 0.001 (pure SWCNTs) to only 0.069 ± 0.012 (KMnO_4_/SWCNTs = 7.5). Thus, when the duration of the process was short (t = 0 min), and the temperature was low, the use of the oxidant was ineffective even when it was present in large amounts. Conversely, when the temperature of the process was increased to 18°C, a rapid increase in kinetics was observed even for the short reaction time. Once the ratio of KMnO_4_/SWCNTs = 1.9 was reached, a notable amount of oxygen-containing functional groups was attached to the SWCNT side walls (I_D_/I_G_ = 0.102 ± 0.033). Beyond this point, the reaction progressed rapidly, eventually stabilizing at I_D_/I_G_ = 0.624 ± 0.006 for KMnO_4_/SWCNTs = 7.5.

**Figure 2:**
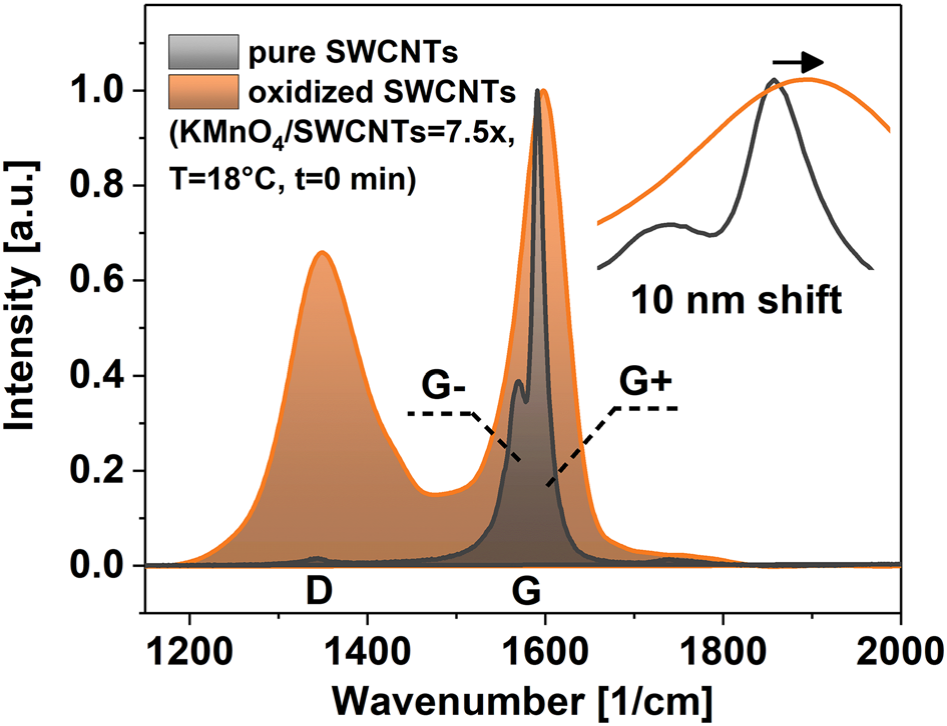
Raman spectra of pure SWCNTs and the oxidation product obtained using the specified conditions. The inset shows a magnification of the G peak area.

As an example, a Raman spectrum of the product obtained under these conditions (KMnO_4_/SWCNTs = 7.5, T = 18 °C, t = 0 min) is shown in Fig. 2. Both the D and the G peaks show considerable broadening upon oxidation. Grafting of functional groups causes bond in-homogeneity and shortening of the lifetime of phonons for the G band^46^. For highly functionalized SWCNTs, the FWHMs of the D and the G band increased extensively to the point that these features started to overlap. Moreover, the treatment affected the G band (previously split into G- and G + components caused by a symmetry breaking of the C–C bond stretching due to the influence of SWCNT curvature^47^). Upon oxidation, these two features could no longer be resolved similarly as in the previous report documenting excessive oxidation of SWCNTs^48^.

Furthermore, it was observed that the position of the G band maximum shifted by as much as 10 cm^−1^ toward higher wavenumbers. Firstly, interfacing SWCNTs with Brønsted acids already p-dopes the nanocarbon due to simple physical interactions^26^. Mineral acids such as H_2_SO_4_ and H_3_PO_4_ used in the study can extract electrons from the system, thereby decreasing its Fermi level^49^. Secondly, the inclusion of oxygen-containing functional groups such as carboxyl and hydroxyl on the SWCNT surface decreases the electron density further due to their electron-withdrawing capabilities^36,50^. Both of these modifications can explain the notable shift highlighted above. The magnitude of the observed shift is quite substantial as SWCNTs are already p-doped in the air due to the presence of oxygen^51^.

**Figure 3:**
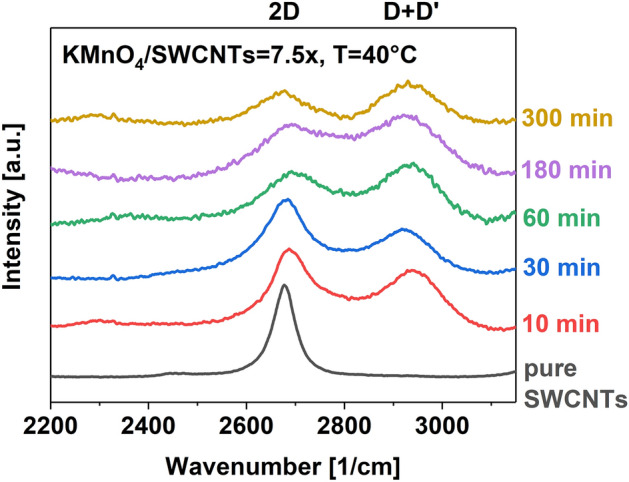
The evolution of 2D and D + D features for pure SWCNTs and the oxidation products carried out using the specified conditions.

Thirdly, an indication of sizeable functionalization of the material was manifested by the emergence of the D + D' feature (Fig. 3) from a two-phonon interband transition facilitated at high defect concentration^46,52^. As shown for the samples obtained at 40 °C, as the intensity of the D + D' peak grew, the intensity of the 2D band gradually decreased due to damages to the SWCNT sidewall.

It is important to stress that a simple exposure of SWCNTs to H_2_SO_4_/H_3_PO4 mixture did not deteriorate the sample as the I_D_/I_G_ ratio remained unchanged (Fig. S2). However, a shift to the G + component was evident, confirming that p-doping did take place. Because the acid-exposed sample was washed with water to neutralize it for analysis, the shift was not significant.

**Figure 4:**
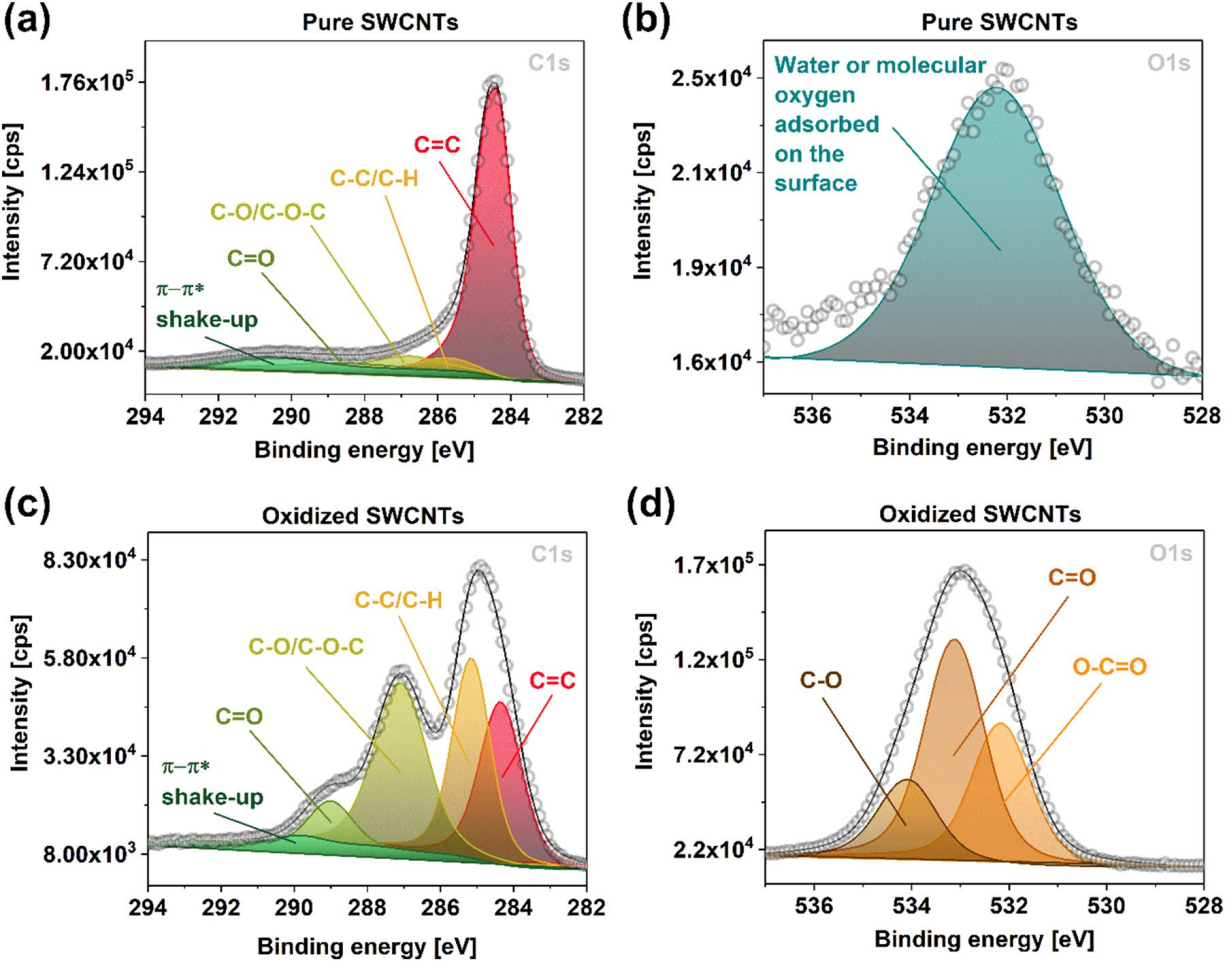
XPS spectra (C1s and O1s areas) of pure SWCNT film and after the treatment at KMnO_4_/SWCNT = 7.5x (T = 18 °C and t = 0 min).

Because XPS gives a more accurate description of functional groups on the surface^53^, it was employed herein (Fig. 4) for two model samples: pure SWCNT film and the film after the oxidative treatment for a short time but at a high oxidant amount (KMnO_4_/SWCNT = 7.5x, T = 18 °C and t = 0 min). The unmodified material confirmed high degree of crystallinity as the intensity of the C=C feature was much larger than that of any other peaks (Fig. 4a). Thus, the starting material was well graphitized. Furthermore, the analysis of the O1s region indicated the presence of water or molecular oxygen on the surface (Fig. 4b)^54–56^. In contrast, dramatic changes were noted for the SWCNT film exposed to the oxidation conditions mentioned above. The intensity of carbon atoms of sp3 type and that of various oxygen-containing functional groups increased considerably (Fig. 4c). Simultaneously, the C=C feature intensity was greatly reduced. Finally, corresponding features were also detected in the O1s spectra of the material (Fig. 4d) revealing the abundance of carboxyl, carbonyl and hydroxyl groups on the surface^57^.

**Figure 5:**
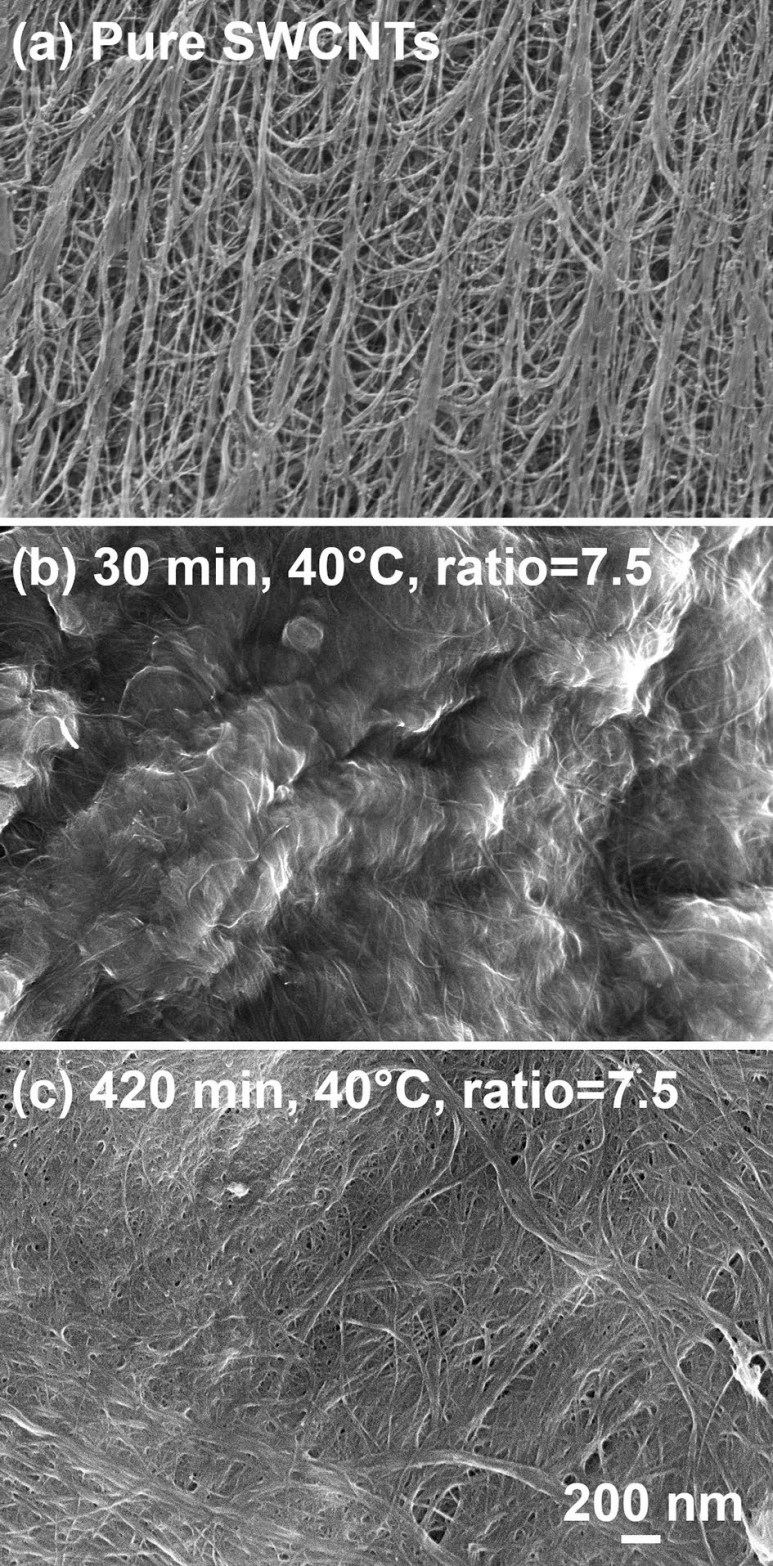
SEM micrographs of pure and oxidized SWCNTs carried out using the specified conditions.

SEM was used to visualize the changes to the microstructure of the material (Fig. 4). Again, the sample obtained at 40 °C processing was analyzed since the oxidation was most dynamic for this temperature. The unmodified SWCNT film was composed of bundles of SWCNTs arranged isotropically as the used drop-casting method of ensemble fabrication does not induce horizontal alignment. Voids are evident in the micrograph (Fig. 5a), which lower the material's capabilities to transport charge. However, the structure became very much densified once the SWCNT film was put in contact with the highly acidic and oxidative medium (Fig. 5b,c). We previously observed that porous CNT films or fibers can compact when the material is exposed to HCl, H_2_SO_4,_ or HNO_3_^58^. When the individual CNTs and their bundles come closer together, the electrical conductivity of the material increases due to simple geometric reasons besides doping^59^. We witnessed the same phenomenon in this study, which is visualized in the SEM micrographs given below.

Furthermore, after 30 min of oxidation, the structure of the SWCNT film was somewhat coated (Fig. 5b), which was not apparent in the micrograph obtained after a longer oxidation time of 420 min (Fig. 5c). This result supports our earlier hypothesis that initially, a part of the material (supposedly defected and small-diameter SWCNTs) was heavily oxidized. Then, as the treatment was continued, these species were removed while the more stable SWCNTs underwent steady functionalization.

### Characterization of ensembles made from oxidized SWCNTs

**Figure 6:**
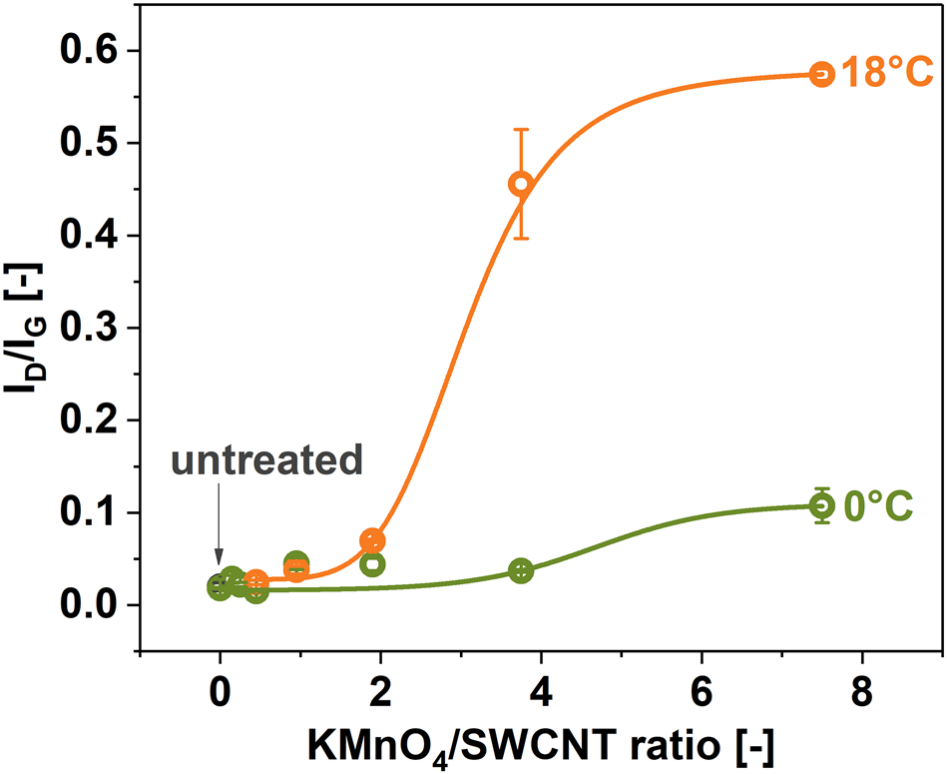
The impact of SWCNT oxidation on the I_D_/I_G_ ratio of the films made from oxidized SWCNTs determined by Raman spectroscopy as a function of KMnO_4_/SWCNT ratio (t = 0 min).

We continued the study by manufacturing and analyzing SWCNT films composed of oxidized SWCNTs. Figure 6 presents the I_D_/I_G_ ratios calculated based on acquired Raman spectra of films made from SWCNTs oxidized at different KMnO_4_/SWCNT ratios at 0 °C and 18 °C. The level of disorder in the SWCNT ensembles increased with the oxidant amount analogously as for the base SWCNT powder, which was used to form SWCNT networks (Fig. 1b). While the I_D_/I_G_ ratio increased from 0.015 ± 0.001 to only 0.108 ± 0.018 at the ratio of KMnO_4_/SWCNT of 7.5 at 0 °C, when the temperature was increased to 18 °C, the I_D_/I_G_ ratio reached as much as 0.574 ± 0.006. Increasing the oxidation temperature makes incorporating functional groups into the SWCNTs much more dynamic. One may note that the recorded values for the films are slightly lower than for the parent SWCNT material used to make them. This is because high-temperature annealing used to remove the EC binder may detach a part of the functional groups from the SWCNT side wall^60^, decreasing the I_D_/I_G_ ratios. The trends are nevertheless preserved.

**Figure 7:**
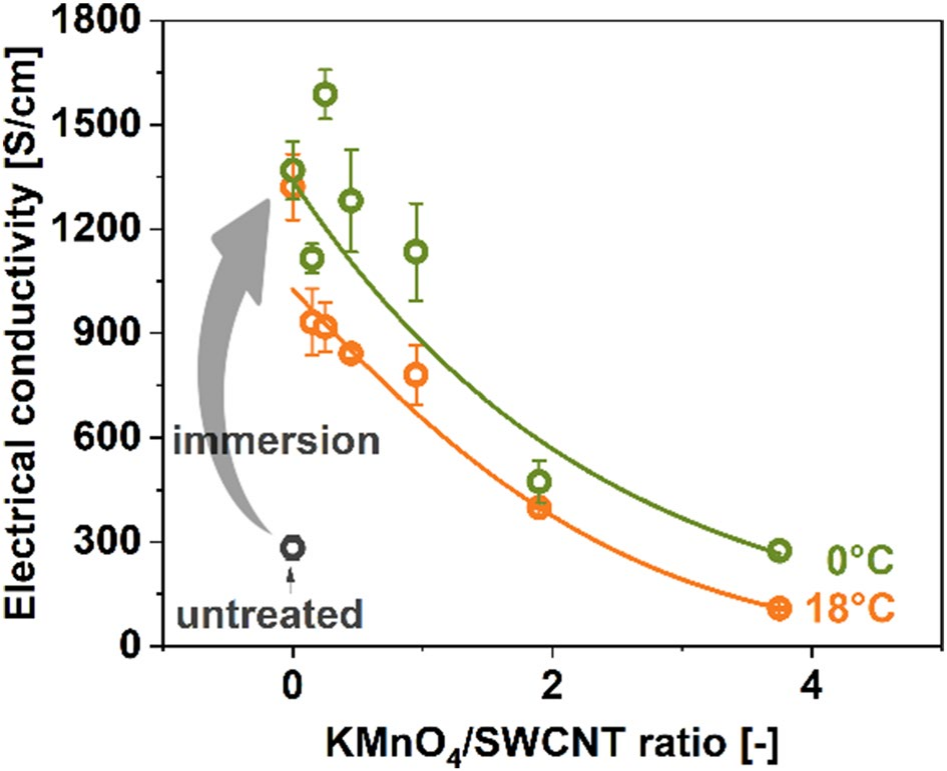
The impact of SWCNT oxidation on the electrical conductivity of the films made from oxidized SWCNTs as a function of KMnO_4_/SWCNT ratio (t = 0 min).

Furthermore, we wanted to find out if it is possible to correlate the degree of structural disorder in the material with its ability to conduct current. Electrical conductivity was established for specimens of oxidized SWCNTs obtained at 0 °C and 18 °C at the selected KMnO_4_/SWCNT ratios (Fig. 7). A considerable enhancement to the electrical conductivity was observed upon immersion of SWCNTs in the reaction mixture not containing KMnO_4_. While the pure SWCNT films had the conductivity of 283 ± 32 S/cm, they reached 1321 ± 95 and 1369 ± 84 S/cm for the treatments at 18 °C and 0 °C at KMnO_4_/SWCNT = 0, respectively. Furthermore, even for the low KMnO_4_/SWCNT ratios, the electrical conductivity values were very much increased due to the doping effect of Brønsted acids (H_2_SO_4_ and H_3_PO_4_)^26^. Thus, under these conditions, the doping phenomenon dominated. As more disorder was introduced to the SWCNTs due to the application of higher amounts of KMnO_4_ oxidant, the electrical conductivity of SWCNT films rapidly decreased.

It has to be noted that strong oxidation conditions had to be established to start the functionalization, but once defects were created, the functionalization continued, as previously discussed. In our experience, the raw SWCNTs used for this study readily withstand oxidation even when put in contact with concentrated HNO_3_ at elevated temperatures. In the modified Hummers method used herein, H_3_PO_4_ and KMnO_4_ are additionally employed to create sufficiently potent oxidizing species. Maciejewska et al. showed that the energy needed to implant a defect such as a methyl group to the surface of (8,0) SWCNT is 4.39 eV. The implementation of an additional 0.62 eV transforms methyl moiety into formyl and subsequently to carboxyl upon the application of a further 0.26 eV. Thus, the oxidation of defects is favored rather than the generation of new ones^61^.

The results enclosed above showed that a small amount of KMnO_4_ did not deteriorate the electrical conductivity much. However, with the increased amount of KMnO_4,_ the functionalization accelerated, which decreased the electrical conductivity of the SWCNT films. Only at the ratios of KMnO_4_/SWCNT > 2, the electrical conductivity of the SWCNT films decreased below the starting value. As expected, the oxidation was more dynamic in the case of reaction carried out at 18 °C due to the faster kinetics of the process.

**Figure 8:**
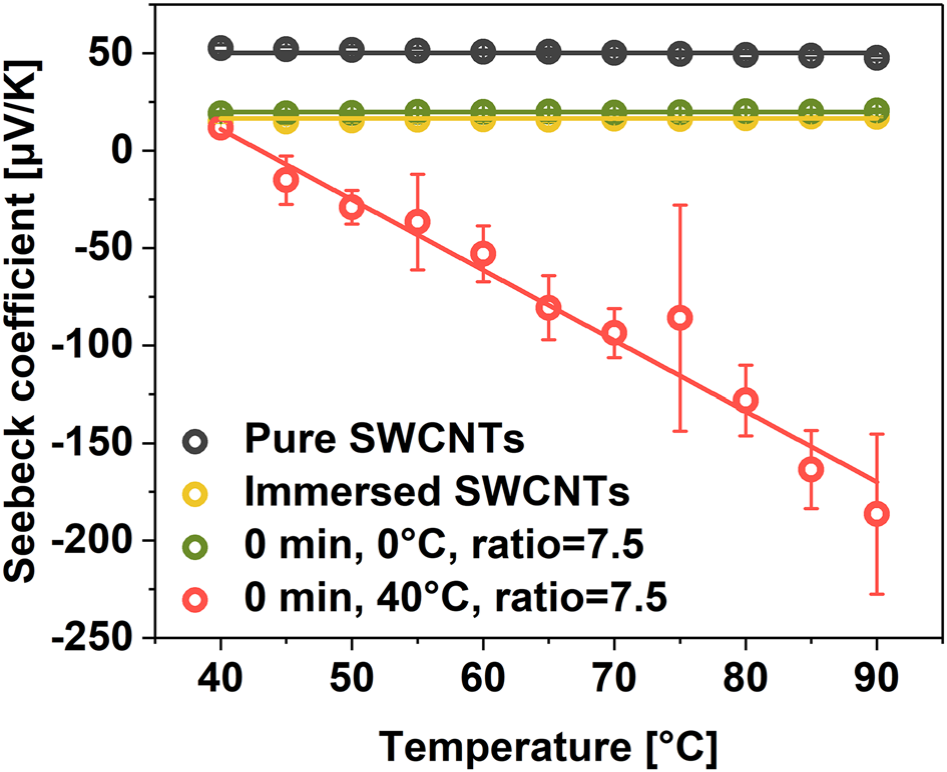
Seebeck coefficient of the described SWCNT materials.

Regardless of the employed KMnO_4_/SWCNT ratio, the treatment significantly affected the material's electrical conductivity. Thus, we decided to analyze the charge transport characteristics of the oxidized SWCNTs/EC composite films by measuring the values of electrical conductivity and Seebeck coefficients for the strongly oxidized samples (KMnO_4_/SWCNT = 7.5, Fig. 8). Interestingly, the electrical conductivity of the SWCNTs oxidized at elevated temperature (40 °C) in the presence of a high amount of KMnO_4_ was decreased steeply. It reached the value of 0.036 ± 0.008 S/cm, 4 orders of magnitude lower than the pure SWCNTs. The characterization of the Seebeck coefficient of this specimen confirmed substantial changes to the electronic nature of the material as it reached a very high absolute value of nearly −200 µV K^−1^ at 90 °C. The changes appeared considerable compared with the starting material exhibiting a Seebeck coefficient of ca. 48 µV/K.

As a reference, we also compared these results against specimens prepared by (a) simple immersion of SWCNTs in H_2_SO_4_/H_3_PO_4_ medium without KMnO_4_ or (b) short exposure to oxidizing conditions (0 min, 0 °C, KMnO_4_/SWCNTs = 7.5). In both cases, the Seebeck coefficients decreased slightly to about 15 µV K^−1^. As mentioned before, mineral acids change the Fermi level of the system by doping, thereby affecting the mobility and density of charge carriers. Excessive carrier density negatively affects the Seebeck coefficient for these samples, which explains the results displayed in Fig. 8 for these two SWCNT film types^62^. To sum up, the results of thermoelectric characterization of all three materials give evidence that the electronic properties of SWCNT films can be tailored by proper selection of oxidation conditions by using the modified Hummers method.

Lastly, we evaluated how the described processing strategy affects the wettability of the material. Previously, we showed that annealing of the free-standing films from CNTs makes them hydrophilic due to the desorption of contaminants from the surface^22,63^. Because we used analogous annealing for this study, it would not be possible to gauge the impact of oxidation by analyzing annealed films from oxidized SWCNTs. Firstly, all SWCNT films would be highly hydrophilic upon thermal removal of the EC binder. Secondly, the annealing step could strip some functional groups from the surface^50^. Considering all these arguments, for this experiment, we manufactured SWCNT films from oxidized material by vacuum filtration through a PTFE membrane, analogously as in the previous paper, to avoid the use of EC binder and annealing^64^. A reference film from unmodified SWCNTs was prepared in the same way.

**Figure 9:**
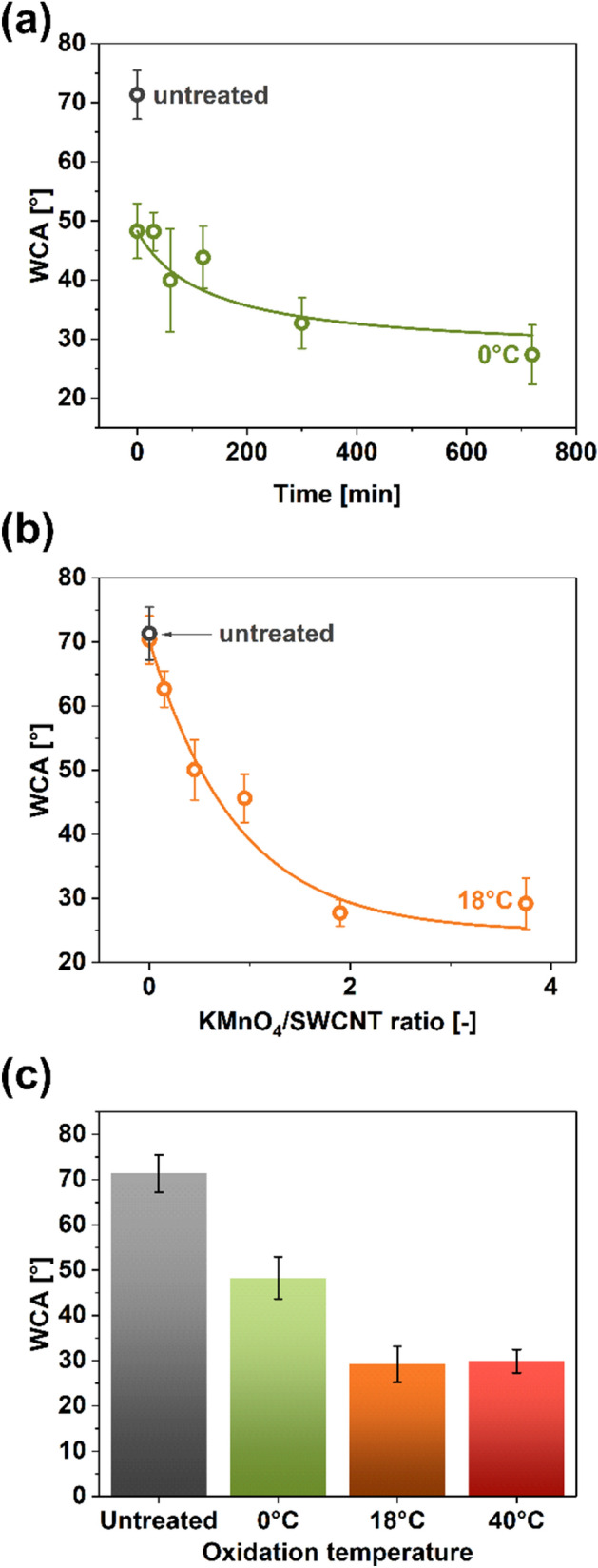
WCA as a function of **(a)** time (T = 0 °C, KMnO_4_/SWCNT = 7.5), **(b)** KMnO_4_/SWCNT ratio (T = 18 °C, t = 0 min), and **(c)** temperature (KMnO_4_/SWCNT = 7.5, t = 0 min).

Figure 9 presents the impact of selected oxidation parameters on the material's static water contact angle (WCA, γ). Firstly, the influence of reaction time was studied at 0 °C and the KMnO_4_/SWCNT ratio of 7.5 (Fig. 9a). The registered WCA gradually decreased with treatment time, which stayed in accordance with the results of Raman spectroscopy. Raman spectra showed that the content of defects increased by a factor of six when the oxidation time was increased to 420 min (Fig. 6), which explains why the nanocarbon becomes more hydrophilic.

Furthermore, the wettability of SWCNT film was affected even at short reaction times and low temperatures. For example, for t = 0 min, the WCA decreased from 71° to 48° with respect to unmodified the SWCNT film. High KMnO_4_ content promoted the changes in microstructure and chemical composition already at 0 °C. As the oxidation time was prolonged, WCA reached 27° after 720 min of the treatment. Under these treatment conditions, not many functional groups could be further incorporated into the SWCNT lattice, so the WCA value leveled off. As previously discussed, the processing at 0 °C utilizes KMnO_4_ ineffectively, so we can conclude that no more reactive oxygen species were available to modify the SWCNTs further.

As expected, the increase in KMnO_4_/SWCNT ratio accelerated the oxidation process, making the SWCNT films more hydrophilic (Fig. 9b). Two findings can be drawn from this experiment. Firstly, simple immersion of SWCNT film in the acid mixture not containing the KMnO_4_ oxidant does not affect the WCA. Secondly, at high KMnO_4_ amounts but short reaction times, similar WCA values may be reached when the treatment is conducted at 0 °C for a long time of 720 min (29° vs. 27°). Thus, it appears that a comparable terminal degree of hydrophilicity of SWCNT films can be obtained by employing the modified Hummers method using different parameters.

Figure 9c compares the impact of oxidation temperature on the WCA of the SWCNT networks. In this case too, once a certain level of hydrophilicity is reached, it cannot be further increased. This is demonstrated by nearly indistinguishable values of WCA for the treatments at 18 °C and 40 °C (taking into account the error bars). Nevertheless, the presented results show that the surface character of the SWCNT films can be straightforwardly tuned by changing the operating conditions of the oxidation of the material by the modified Hummers method.

It should be kept in mind that contact angle values are affected by roughness and surface condition^65^. Since these factors were not considered herein, the wettability results demonstrated above should only be used qualitatively i.e. they illustrate general trends, while the reported absolute values bear less importance.

## Conclusions

This study has identified the impact of three parameters of the modified Hummers method (time, temperature, and KMnO_4_ oxidant amount) on SWCNTs' microstructure, purity, and properties. It was shown that the treatment can tune these characteristics of networks made from SWCNTs. Depending on the severity of the processing conditions, either doping or oxidation prevails.

The conducted research indicated that increasing the oxidation temperature speeds up its kinetics significantly. Moreover, much more imperfection is introduced in the end to the material at high temperatures, even at extended oxidation times. Regarding the temperature component, the results of this study displayed that for the low temperature of 0 °C, defects are steadily introduced into the SWCNTs, thereby making the material more hydrophilic. However, the execution of the treatment at 18 °C and 40 °C revealed slightly different mechanics of this process. Based on these results, we concluded that low-diameter SWCNTs seem to be oxidized more rapidly, whereas the large-diameter fraction is gradually grafted with oxygen-containing functional groups.

Interestingly, either short processing times or the low amount of KMnO_4_ during the treatment gives an SWCNT network of considerably improved electrical properties, which increased from 283 ± 32 to 1369 ± 84 S/cm due to the doping effect. In light of the presented information, the proposed processing routine appears as a powerful tool to tailor the properties of SWCNT networks, such as electrical conductivity and wettability for a specific application. Even when these materials are slightly oxidized, they preserve high capabilities for charge transport. Simultaneously, they become much more compatible with other materials such as polymers (increased water contact angle) at a slight expense of electrical conductivity. Consequently, such a trade-off gives SWCNT networks more ready for utilization in rapidly emerging application areas. For instance, due to the improved wettability, they may be used in conductive composites or charge storage devices. For such implementations, appropriate compatibility with the matrix is equally important as high electrical conductivity.

## Supplementary Information

Supplementary Figures.
