# Supplementary material for: Tuning wettability and electrical conductivity of single-walled carbon nanotubes by the modified Hummers method
Source: Sci Rep. 2022 Mar 14;12:4358. doi: 10.1038/s41598-022-08343-5 (PMC8921219; doi:10.1038/s41598-022-08343-5)
Supplement: Supplementary file 1 — Supplementary Figures. [file 41598_2022_8343_MOESM1_ESM.pdf]

Supplementary Information

Tuning wettability and electrical conductivity of single-walled carbon nanotubes by the modified Hummers method

Grzegorz Stando[1,2,*], Sujie Han[3], Bogumiła Kumanek[1,4], Dariusz Łukowiec[5], Dawid Janas[1,*]

[1] Department of Organic Chemistry, Bioorganic Chemistry and Biotechnology, Faculty of Chemistry, Silesian University of Technology, B. Krzywoustego 4, 44-100 Gliwice, Poland

[2] Department of Chemistry, University of Pittsburgh, Pittsburgh PA, USA

[3] School of Materials Science and Chemical Engineering, Ningbo University, 818 Fenghua Road, Ningbo City, P.R. China

[4] Laboratory of Material Engineering and Environment, KOMAG Institute of Mining Technology, 44-101 Gliwice, Poland

[5] Institute of Engineering Materials and Biomaterials, Faculty of Mechanical Engineering, Silesian University of Technology, Konarskiego 18, 44-100 Gliwice, Poland

Corresponding authors: Grzegorz.Stando@polsl.pl, Dawid.Janas@polsl.pl

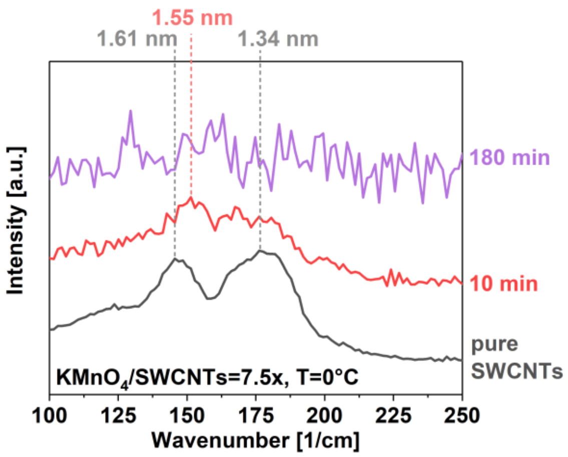

Figure S1 Analysis of the RBM area of the Raman spectra recorded for pure SWCNTs and the oxidation products obtained using the specified conditions.

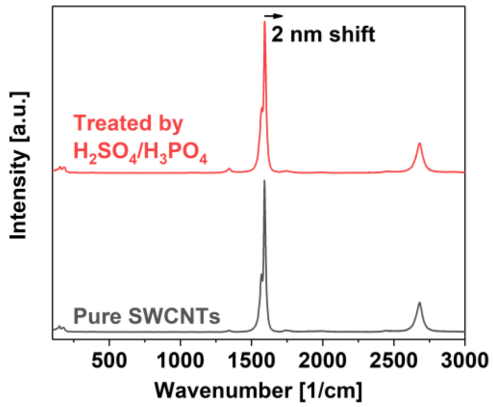

Figure S2 Analysis of the Raman spectra recorded for pure SWCNTs and after immersion in H_2SO_4/H_3PO_4 mixture.
